# Quality, Features, and Presence of Behavior Change Techniques in Mobile Apps Designed to Improve Physical Activity in Pregnant Women: Systematic Search and Content Analysis

**DOI:** 10.2196/23649

**Published:** 2021-04-07

**Authors:** Melanie Hayman, Kristie-Lee Alfrey, Summer Cannon, Stephanie Alley, Amanda L Rebar, Susan Williams, Camille E Short, Abby Altazan, Natalie Comardelle, Sinead Currie, Caitlin Denton, Cheryce L Harrison, Tayla Lamerton, Gabriela P Mena, Lisa Moran, Michelle Mottola, Taniya S Nagpal, Lisa Vincze, Stephanie Schoeppe

**Affiliations:** 1 School of Health, Medical and Applied Sciences CQUniversity Rockhampton Australia; 2 School of Psychological Sciences University of Melbourne Melbourne Australia; 3 Pennington Biomedical Research Center Louisiana State University Baton Rouge, LA United States; 4 Division of Psychology Stirling University Scotland United Kingdom; 5 School of Public Health and Preventive Medicine Monash University Melbourne Australia; 6 School of Human Movement and Nutrition Sciences University of Queensland Brisbane Australia; 7 Faculty of Health Sciences in Kinesiology University of Western Ontario London, ON Canada; 8 School of Human Kinetics University of Ottawa Ottawa, ON Canada; 9 School of Allied Health Sciences - Nutrition and Dietetics Griffith University Gold Coast Australia

**Keywords:** pregnancy, exercise, physical activity, mobile health (mHealth), applications, MARS, behavior change techniques, mobile phone

## Abstract

**Background:**

Physical activity during pregnancy is associated with several health benefits for the mother and child. However, very few women participate in regular physical activity during pregnancy. eHealth platforms (internet and mobile apps) have become an important information source for pregnant women. Although the use of pregnancy-related apps has significantly increased among pregnant women, very little is known about their theoretical underpinnings, including their utilization of behavior change techniques (BCTs). This is despite research suggesting that inclusion of BCTs in eHealth interventions are important for promoting healthy behaviors, including physical activity.

**Objective:**

The aim of this study was to conduct a systematic search and content analysis of app quality, features, and the presence of BCTs in apps designed to promote physical activity among pregnant women.

**Methods:**

A systematic search in the Australian App Store and Google Play store using search terms relating to *exercise* and *pregnancy* was performed. App quality and features were assessed using the 19-item Mobile App Rating Scale (MARS), and a taxonomy of BCTs was used to determine the presence of BCTs (26 items). BCTs previously demonstrating efficacy in behavior changes during pregnancy were also identified from a literature review. Spearman correlations were used to investigate the relationships between app quality, app features, and number of BCTs identified.

**Results:**

Nineteen exercise apps were deemed eligible for this review and they were accessed via Google Play (n=13) or App Store (n=6). The MARS overall quality scores indicated moderate app quality (mean 3.5 [SD 0.52]). Functionality was the highest scoring MARS domain (mean 4.2 [SD 0.5]), followed by aesthetics (mean 3.7 [SD 0.6]) and information quality (mean 3.16 [SD 0.42]). Subjective app quality (mean 2.54 [SD 0.64]) and likelihood for behavioral impact (mean 2.5 [SD 0.6]) were the lowest scoring MARS domains. All 19 apps were found to incorporate at least two BCTs (mean 4.74, SD 2.51; range 2-10). However, only 11 apps included BCTs that previously demonstrated efficacy for behavior change during pregnancy, the most common being *provide opportunities for social comparison* (n=8) and *prompt self-monitoring of behavior* (n=7). There was a significant positive correlation between the number of BCTs with engagement and aesthetics scores, but the number of BCTs was not significantly correlated with functionality, information quality, total MARS quality, or subjective quality.

**Conclusions:**

Our findings showed that apps designed to promote physical activity among pregnant women were functional and aesthetically pleasing, with overall moderate quality. However, the incorporation of BCTs was low, with limited prevalence of BCTs previously demonstrating efficacy in behavior change during pregnancy. Future app development should identify and adopt factors that enhance and encourage user engagement, including the use of BCTs, especially those that have demonstrated efficacy for promoting physical activity behavior change among pregnant women.

## Introduction

Physical activity during pregnancy is associated with a variety of health benefits, including reduced risk of excessive gestational weight gain, gestational diabetes, gestational hypertension, preeclampsia, the severity of pelvic girdle pain, macrosomia, instrumental delivery, postpartum weight retention, urinary incontinence, and depressive disorders [1,2]. Despite the many health benefits of physical activity during pregnancy, few women participate in regular physical activity during pregnancy [3]. In addition, women tend to reduce or cease their participation in physical activity once they become pregnant and throughout the course of their pregnancy [3,4]. This may be as a result of various barriers, including mother-child safety concerns, fatigue, change in body shape, and associated pain. Further, a lack of provision of adequate information, knowledge, social support, and self-efficacy for behavior change are other issues that may exacerbate the decline in activity levels throughout pregnancy [2,5].

There are many avenues for women to access information and support related to maintaining a healthy pregnancy, including information about physical activity behaviors. Historically, pregnant women have accessed information from doctors, midwives, family, and friends to guide and inform their physical activity behaviors. However, eHealth platforms such as the internet and mobile apps are now altering the way women access this information [6] and they have become an important information source for pregnant women [7,8]. In fact, a recent Australian study among 410 pregnant women investigated the use of pregnancy and parenting apps and found that almost three-quarters of the studied women used at least one of these types of pregnancy apps [9]. In addition, more than half of the participants reported using 2-4 apps throughout their pregnancy. The frequency of app use was also significant, with almost a quarter of pregnant women reporting daily use of apps [9]. While the use of pregnancy-related apps has significantly increased among pregnant women [8], very little is known about their theoretical underpinnings, including their utilization of behavior change techniques (BCTs). This is despite research suggesting that inclusion of BCTs in eHealth interventions can play an important role in improving, supporting, and maintaining healthy behaviors, including physical activity [10,11].

In 2013, Currie et al [12] systematically evaluated the content of physical activity interventions designed to reduce the decline of physical activity in pregnant women with a specific emphasis on BCTs [13] employed to elicit this change. Six common BCTs shown to have some efficacy in improving physical activity behaviors were identified: prompt intention formation, prompt specific goal setting, prompt review of behavioral goals, prompt self-monitoring of behavior, provide feedback on performance, and provide opportunities for social comparison [12]. Since this review, many behavior change interventions have used these BCTs to promote positive physical activity behaviors among pregnant women [14].

Previous reviews of physical activity apps for other population groups suggest that commercial apps often lack evidence-based BCTs that have demonstrated efficacy for encouraging physical activity behavior change [15-17]. However, no such review of commercial apps designed to promote physical activity among pregnant women has been conducted. Thus, the appropriateness of these apps to promote physical activity during pregnancy is unknown. This review aimed to systematically evaluate the appropriateness of the apps designed to promote physical activity among pregnant women by using a systematic search and content analysis. Apps available through the Australian App Store and Google Play stores were accessed using the MARS tool for app quality and features. A taxonomy of BCT was also used to assess the presence of BCTs utilized within the apps, including BCTs that have demonstrated efficacy for promoting physical activity behavior change among pregnant women.

## Methods

### Methodological Approach

The methodological approach used in this study was informed by previous app reviews. These reviews explore app quality, features, and BCTs among apps designed to (1) improve diet, physical activity, and sedentary behavior in children and adolescents [15] and (2) provide nutritional advice to pregnant women [18].

### Search Strategy

Systematic searches were conducted in the Australian App Store and Google Play stores between October 2018 and February 2019. Apps were identified using systematic combinations of the following search terms: pregnancy, pregnant, prenatal, postnatal, exercises, exercise, fitness, workout, and physical activity. These search term combinations were entered individually in the App Store and Google Play databases without any specified search categories, and search results were ordered by relevance (see Multimedia Appendix 1). As Google Play search results were capped at 250 apps, only the title and description of the first 250 relevant apps (in Google Play and App Store) were screened.

### Inclusion Criteria and Selection Process

Apps were considered for inclusion if the description of the app in the stores specified pregnancy content and physical activity or exercise. Apps were included if they (1) targeted pregnant women, (2) had a focus on physical activity or exercise, (3) were available in English, and (4) had a user rating of at least 4.5 (scale range 1-5) in either of the stores (as similarly done elsewhere [17]) as a measure of app popularity. Both free and paid apps were eligible for inclusion; however, apps requiring external devices (eg, Kegel device, activity monitor, hardcopy books) were excluded due to limitations regarding device access and use. App selection and assessments were undertaken between October 2018 and April 2019.

As per best practice for systematic reviews [19], 2 reviewers (KLA and SC) independently reviewed the titles, images, and descriptions of each identified app for inclusion in the review. Disagreement was resolved by discussion and consensus with a third reviewer (MH). Each of the eligible apps were examined independently by 2 of the 18 reviewers (ie, the authors), who were recognized as having expertise in behavior change or physical activity during pregnancy. If there was any notable disagreement in variance (eg, disagree versus agree) among the app assessment scores, a third reviewer would also be assigned. Each reviewer was allocated 4-6 apps to examine, determined by device accessibility (Apple or Android). Examination of apps included downloading, user testing, and assessing app features and quality criteria. Each app was allocated to an expert in behavior change and to an expert in physical activity during pregnancy for review. Incorporation of BCTs within each app were independently reviewed by 2 reviewers (KLA and SC). Any disagreements/discrepancies between reviewers KLA and SC were resolved by consultation with a third reviewer (MC). If an app was available in both App Store and Google Play, either version could be utilized for testing, regardless of differences in app user ratings. To maintain a consistent cost status and baseline assessment, if an app offered a free version and a paid version, the free version was included. To maintain this consistency, freemium content (ie, extra content at a cost) was not accessed and apps requiring paid subscriptions were excluded.

### Data Extraction

Data extraction was conducted using a standard information spreadsheet and the Mobile App Rating Scale (MARS) [20]. Similar methods have been utilized in previous app reviews [15,21]. For all included apps, app name, developer, version, store (App Store, Google Play), cost (free, paid), average user rating (at least 4.5+), MARS focus points (what the app targets, eg, increase happiness/well-being, behavior change, entertainment, physical health), MARS theoretical background/strategies (eg, assessment, information/education, goal setting, advice/tips/strategies/skills training), and MARS technical aspects (eg, allows sharing, allows password protection, sends reminders) were extracted (see Multimedia Appendix 2 and Multimedia Appendix 3).

### App Features and Quality Assessment

App features and quality were assessed using the MARS [20], as per prior app reviews [15,22]. The MARS consists of 19 items grouped in 4 domains: engagement (entertainment, interest, customization, interactivity, and target group); functionality (performance, ease of use, navigation, and gestural design); aesthetics (layout, graphics, and visual appeal); and information quality (accuracy of app description, goals, quality, and quantity of information, visual information, credibility, and evidence base). Additional MARS domains of subjective app quality (recommendation, potential use, payment, and overall rating) and likelihood of behavioral impact (awareness, knowledge, attitudes, intention to change, help seeking, and behavior change) were also included. Items were measured on a 5-point scale (1=inadequate to 5=excellent) and a score for each domain was computed as the mean of the items in that domain; the overall score was computed as an average across the domains [20]. Final scores for each app and MARS items were calculated using the means of the reviewer scores (see Multimedia Appendix 4).

### BCT Identification

The assessment of the presence or absence of BCTs for improving physical activity behavior was guided by the taxonomy of BCTs developed by Abraham and Michie [13]. A dichotomous score of 0 (absent) or 1 (present) was applied for each of the 26 BCTs, resulting in a total score of 0-26 (see Multimedia Appendix 5). This approach has been applied in similar app reviews and content analyses [15,23,24].

### Identification of Evidence-Based BCTs

A brief literature search was employed to understand the BCTs that may be effective in supporting behavior change during pregnancy. A systematic review by Currie et al [12] described 6 BCTs that hold efficacy in reducing the decline of physical activity among pregnant women. These BCTs include prompt intention formation, prompt specific goal setting, prompt review of behavioral goals, prompt self-monitoring of behavior, provide feedback on performance, and provide opportunities for social comparison*.* As pregnancy-effective BCTs, these were specifically highlighted during analysis and results.

### Statistical Analyses

In addition to descriptive statistics (mean, standard deviation, and range) calculated for each of the 6 MARS domains, frequencies (numbers and percentages) of each of the 26 BCTs included in the apps were calculated. Krippendorff’s alpha (Kα) was used to evaluate interrater reliability for the app quality assessment and the presence of BCTs within the apps [25]. Spearman correlations were used to examine the relationships between app quality, number of technical app features, and number of BCTs incorporated in the apps. All statistical analyses were conducted using SPSS Statistics version 26.0 (IBM Corp) with significance levels set at *P*<.05.

## Results

### App Selection

**Figure 1:**
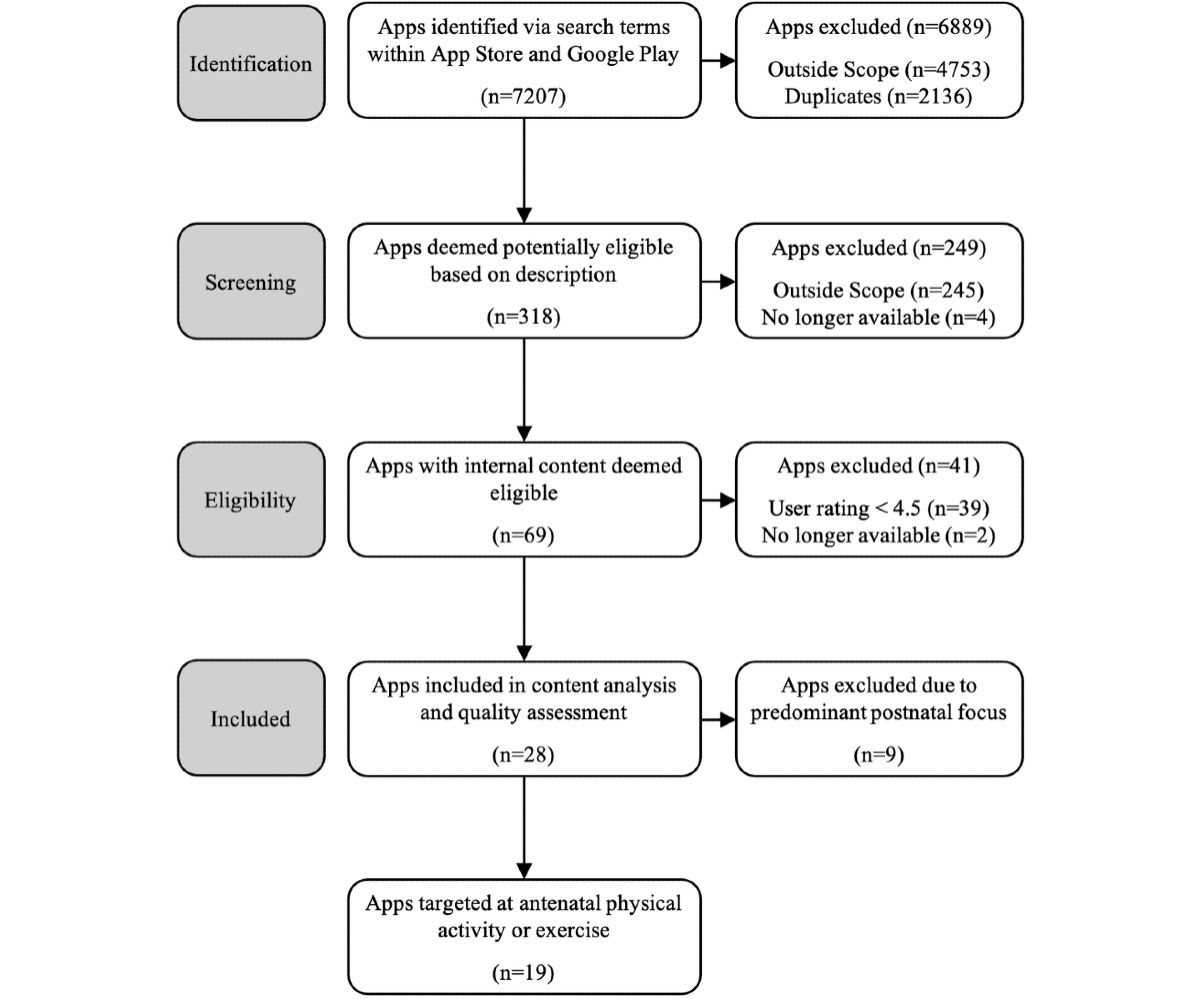
PRISMA flow chart of the app selection process.

A flowchart of the app selection process is presented in Figure 1. A total of 7207 apps were identified and screened in the App Store and Google Play. Of these, 318 apps were further screened by description and 69 apps held content considered eligible for inclusion. The user rating criteria of 4.5+ was applied and apps found to focus solely on postnatal physical activity/exercise were omitted. A total of 19 apps targeting physical activity during pregnancy were included in the content analysis and quality assessment.

### App Characteristics

**Figure 2:**
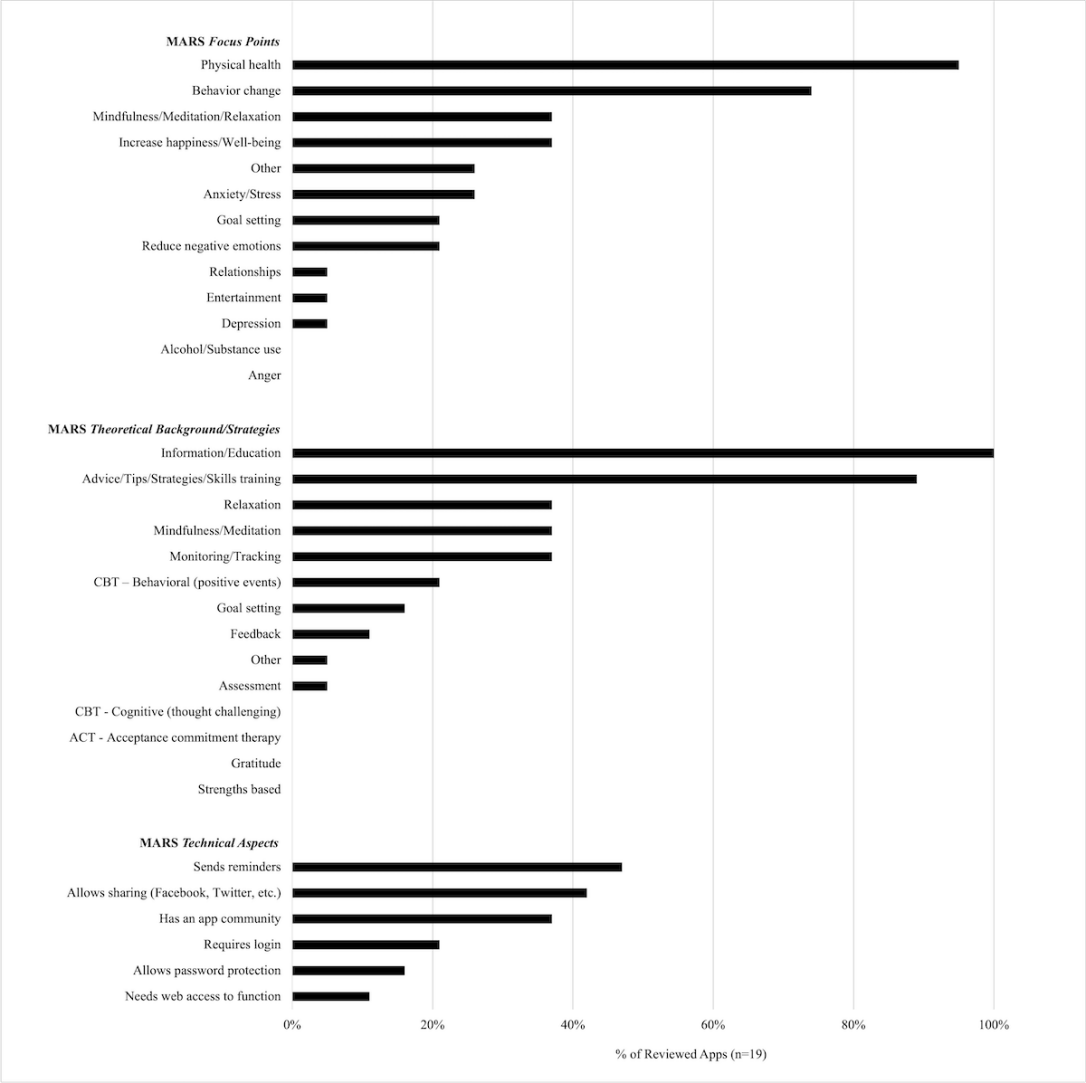
Categories of focus points, theoretical background and strategies, and technical aspects of the Mobile App Rating Scale found in each app.

Of the 19 reviewed antenatal physical activity apps, 13 were accessed via Google Play and 6 were accessed via App Store (see Multimedia Appendix 2). Apps were free to download, with the exception of one. The average star rating for the apps was 4.69 (SD 0.22), with a wide range of the number of users rating each app (mean 1875.16, SD 3549.82; range 1-13,000). On average, the 19 apps were found to contain few MARS-related categories: MARS focus points (mean 3.53 [SD 1.90]), MARS theoretical background/strategies (mean 3.58 [SD 1.98]), and MARS technical aspects (mean 1.74 [SD 1.73]). Figure 2 and Multimedia Appendix 3 detail the MARS categories for each of the 19 apps.

### App Quality

The average MARS overall quality score was 3.5 out of 5 with a range of 2.4-4.3, which was considered to be of moderate quality. Functionality was the highest scoring domain (mean 4.2 [SD 0.5], followed by aesthetics (mean 3.7 [0.6]), information quality (mean 3.16 [SD 0.42]), and engagement (mean 3.01 [SD 0.9]). Subjective app quality (mean 2.5 [SD 0.6]) and likelihood for behavioral impact (mean 2.5 [SD 0.6]) were equally the lowest scoring MARS domains. Table 1 provides a summary of the MARS scores. A detailed summary of the quality assessment of the included apps is presented in Multimedia Appendix 4. Interrater reliability [25] for app quality resulted in low reliability (mean Kα 0.3, SD 0.37). However, there was no notable disagreement in variance (eg, disagree versus agree) among the app assessment scores; thus, a third reviewer was not required for any further app assessment.

**Table 1:** Summary of the Mobile App Rating Scale (scale 1-5) scores across the 19 reviewed apps.^a^

Mobile App Rating Scale domain	Mean score (SD)	Median score (IQR)	Range of scores (min-max)
Functionality	4.22 (0.49)	4.3 (0.75)	1.7 (3.3-5)
Aesthetics	3.69 (0.64)	4 (0.95)	2.2 (2.3-4.5)
Overall quality	3.52 (0.52)	3.5 (0.7)	1.9 (2.4-4.3)
Information quality	3.19 (0.42)	3.2 (0.75)	1.4 (2.6-4)
Engagement	3.01 (0.9)	3 (1.4)	3.3 (1.2-4.5)
Subjective quality	2.54 (0.64)	2.5 (0.95)	2.3 (1.5-3.8)
Likelihood of behavioral impact	2.54 (0.62)	2.6 (0.6)	2.2 (1.6-3.8)

^a^Score 1=inadequate; score 5=excellent.

### Presence of BCTs

**Figure 3:**
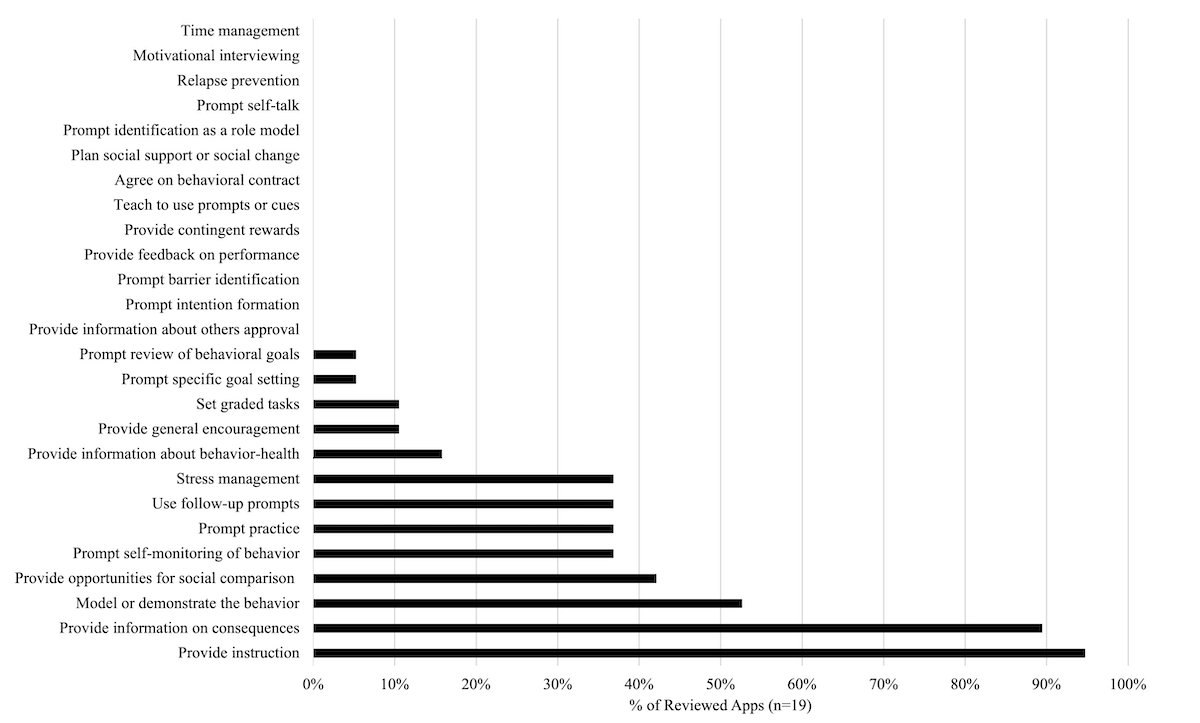
Presence and types of behaviour change techniques found in the selected apps.

The presence and types of BCTs found within the reviewed apps are presented in Figure 3 and Multimedia Appendix 5. Interrater reliability for evaluating the presence of BCTs in the apps was high (Kα 0.85, percent agreement 95%). All reviewed apps incorporated at least two BCTs. Commonly included BCTs included *provide instructions* (18/19, 95%), *provide information on consequences* (17/19, 89%), *model or demonstrate the behavior* (10/19, 53%), and *provide opportunities for social comparison* (8/19, 42%). The average number of BCTs per app was 4.74 (SD 2.51) (range 2-10). Apps with the highest number of BCTs included were Pregnancy Week by Week Tracker (10 BCTs), iMum-Pregnancy & Fertility (9 BCTs), and Pregnancy Tracker & Countdown (9 BCTs).

### Presence of Evidence-Based BCTs

Currie et al [12] identified 6 common BCTs shown to have some efficacy in reducing the decline in physical activity behaviors among pregnant women, namely, *prompt intention formation, prompt specific goal setting, prompt review of behavioral goals, prompt self-monitoring of behavior, provide feedback on performance,* and *provide opportunities for social comparison*. Of the 19 apps reviewed in the present study, 11 apps contained at least one of these evidence-based BCTs (range 1-3) and 4 contained more than one of these evidence-based BCTs. Table 2 details the evidence-based BCTs included in each of the 11 apps.

**Table 2:** Apps containing evidence-based techniques for behavior change during pregnancy.^a^

App name	Evidence-based behavior change techniques					
	Prompt intention formation (n=0)	Prompt specific goal setting (n=1)	Prompt review of behavioral goals (n=1)	Prompt self-monitoring of behavior (n=7)	Provide feedback on performance (n=0)	Provide opportunities for social comparison (n=8)
Pregnancy+ (n=3)			✓	✓		✓
Pregnancy Week by Week Tracker (n=3)		✓		✓		✓
Pregnancy Tracker & Countdown (n=2)				✓		✓
Pregnancy Workouts-Baby2Body (n=2)				✓		✓
9Months Guide (n=1)						✓
Get Parenting Pregnancy Tips. Moms Pregnancy App (n=1)						✓
I’m Pregnant-Pregnancy Tracker (n=1)				✓		
iMum-Pregnancy & Fertility (n=1)				✓		
Kegel Exercises (n=1)				✓		
Pregnancy Guide (n=1)						✓
Pregnancy Health (n=1)						✓

^a^Apps not identified as containing a key behavior change technique are not noted in the table.

### Relationships Between App Quality, App Features, and BCTs

Spearman correlations between the MARS overall quality, number of MARS focus points, number of MARS theoretical background/strategies, number of MARS technical aspects used in the app, MARS subjective quality, and the number of BCTs are presented in Table 3. The number of identified BCTs was positively associated with the MARS engagement score (ρ=0.55, *P*=.01) and aesthetics score (ρ=0.46, *P*=.046). MARS functionality, information quality, total MARS quality, and subjective quality were not significantly correlated with the number of BCTs. The number of app focus points was positively associated with the MARS engagement score (ρ=0.58, *P*=.009), aesthetics score (ρ=0.58, *P*=.008), total MARS quality score (ρ=0.55, *P*=.02), and subjective quality score (ρ=0.46, *P*=.048). The number of technical aspects within apps was positively correlated with the MARS engagement score (ρ=0.63, *P*=.004) but not with any of the other MARS scores. Further, the MARS subjective quality scores were positively correlated with all other quality subscores (Table 3) and the total MARS quality score (ρ=0.90, *P*<.001).

**Table 3:** Correlations between Mobile App Rating Scale (MARS) overall quality, number of MARS focus points, number of MARS theoretical background/strategies, number of technical aspects, and MARS subjective quality.

MARS subscales	Behavior change techniques	MARS focus points	MARS theoretical background/ strategies	MARS technical aspects	MARS subjective quality
MARS engagement	0.55^a^	0.58^b^	0.69^b^	0.63^b^	0.69^b^
MARS functionality	0.02	0.30	0.36	–0.24	0.64^b^
MARS aesthetics	0.46^a^	0.59^b^	0.61^b^	0.37	0.90^b^
MARS information quality	0.32	0.34	0.37	0.10	0.69^b^
MARS overall quality	0.45	0.55^a^	0.67^b^	0.31	0.90^b^
MARS subjective quality	0.42	0.46^a^	0.60^b^	0.36	—^c^

^a^Correlation was significant at *P*<.05.

^b^Correlation was significant at *P*<.01.

^c^Not applicable.

## Discussion

The primary aim of this app review was to assess app quality and features and presence of BCTs applied within commercial apps promoting physical activity in women during pregnancy. The secondary aim of this app review was to test the relationships between the 6 MARS domains of app quality and the number of MARS technical aspects, theoretical strategies, focus points, and BCTs. In summary, our findings demonstrate moderate MARS overall quality scores, with MARS domains of functionality and aesthetics scoring the highest and the domains of subjective app quality and likelihood for behavioral impact scoring the lowest. An average of 4.74 BCTs per app were used, with the most common BCTs being *provide information on consequences* and *provide instructions*. Most apps had none or one of the BCTs that demonstrated efficacy in promoting physical activity behaviors during pregnancy, with the most common BCTs being *opportunities for social comparison* and *prompt self-monitoring of behavior*.

Many commercially available physical activity apps for pregnant women were identified—with 69 relevant apps and 19 apps remaining after apps targeting postnatal physical activity (n=9) and apps with user ratings lower than 4.5 (n=39) were excluded. This finding demonstrates a market for apps targeting physical activity during pregnancy, which is not surprising, considering the findings of Lupton and Pedersen [9] who have previously reported that pregnant women frequently use pregnancy-related apps. The 69 apps identified as targeting exercise in pregnancy is comparable to the 51 apps Brown et al [18] identified as targeting nutrition in pregnancy. Pregnancy seems to be a time in women’s lives in which they are actively seeking and using apps to support their behaviors, including physical activity.

The high MARS functionality scores and moderate-to-high MARS aesthetics scores may be a consequence of developers focusing on user experience for visually pleasing and user-friendly apps [26]. Although such features are important for attracting users, they do not translate to behavior change [27,28]. There is also room for improvement in app design in relation to the low MARS scores for quality of information and engagement. Given that pregnant women believe the information in apps has high credibility [9], it is important that apps ensure that evidence-based information is provided. It is also important that apps improve ratings of engagement, as engagement in app content is associated with behavior change [22].

Ratings of engagement could be improved by increasing the use of BCTs. Our study found an association between the number of BCTs and improved MARS engagement and aesthetics scores, which is consistent with the findings from a review investigating apps for weight management in adults [22]. The association between the number of BCTs and the engagement score could be due to the provision of additional content to engage in, increased usefulness, and perceived efficacy. It may be that BCTs are considered as enticements to potential users, thereby increasing engagement. If this is the case, this provides additional incentives for app developers to include BCTs. In line with that reported by Schoeppe et al [15] and Bardus et al [22], this study also found technical aspects of health behavior change apps to be associated with the MARS engagement score. Further, the number of theoretical strategies and the number of focus points were associated with MARS engagement, aesthetics, total quality, and subjective quality scores. Improving the number of technical aspects, theoretical strategies, and focus points may therefore help to further improve engagement of apps designed to promote physical activity among pregnant women.

An average of 4.74 BCTs were used in the reviewed apps. This is in line with the findings from previous research that found apps for physical activity promotion in adults to have an average of 4.2 [16] and 5.0 BCTs [24]. The number of BCTs identified in this review is, however, higher than those identified in a recent review exploring the number of BCTs among apps targeting nutrition behaviors in pregnancy [18], which reported an average of 3 BCTs per app [18]. We know that health behavior interventions using alternative modes of delivery (eg, websites) have improved outcomes when more BCTs are used [29]. Webb et al [29] suggest that this improvement may be a result of using a combination of BCTs that together target several stages and aspects of behavior change. However, we do not know the optimal number or combination of BCTs necessary to increase physical activity.

The most common BCTs identified in this study were *provide information on consequences* and *provide instructions*. Schoeppe et al [15] also found that providing instructions was a commonly used BCT in physical activity and diet apps for children and adolescents. This finding differs from the most common BCTs identified in apps targeting weight and physical activity in adults, which are goal setting, self-monitoring, and performance feedbacks [15,29]. Unfortunately, *provide information on consequences* and *provide instructions* have not been shown to be effective at improving physical activity behaviors in pregnant women [12] or even in the general adult population [27].

Most apps in this review had none or 1 BCT, which previously demonstrated efficacy in behavior change during pregnancy. Including more of these evidence-based BCTs in the context of pregnancy may improve the ability of the apps to support pregnant women in increasing their physical activity [12]. The evidence-based BCTs of intention formation, goal setting, review of goals, and feedback on performance have been successfully implemented within physical activity apps targeting other population groups [15,16]. This demonstrates that it is feasible to deliver these evidence-based BCTs through an app. In particular, feedback on performance was identified as one of the most commonly used BCTs in apps targeting physical activity, diet, and sedentary behavior in children and adults [15]. Feedback on performance requires the measurement of behavior (eg, frequency, intensity of exercise), and apps that incorporate wearable devices (eg, Fitbit, Garmin) to measure physical activity behavior may be more likely to provide feedback on performance [30]. Therefore, the exclusion of apps that required the use of a wearable device may partially explain the finding of low use of feedback on performance in this study. The most common technical features were *sends reminders* (9/19, 47%) and *allows sharing* (8/19, 42%). There is evidence to suggest that reminders improve the effectiveness of health behavior change interventions [31]; however, other evidence suggests that reminders can hinder habit formation, which may have an impact on long-term behavior change [32]. The high use of sharing is contradictory to the findings that adults find social media features unnecessary and off-putting in health behavior change apps [33]. The removal of sharing features may improve engagement ratings.

The strengths of this study include the systematic search for apps from both App Store and Google Play, the use of an established taxonomy for identifying BCTs, the use of the MARS instrument to assess the quality of apps, and the inclusion of apps rated above 4.5/5. Further, app ratings were performed by a minimum of two reviewers following best practices for conducting systematic reviews [19]. Good interrater reliability was found for the scoring of BCTs.

The limitations of this study include the exclusion of apps that required use of a wearable device and low interrater reliability for the scoring of app quality through the MARS scale. This may be due to the subjective nature of some sections of the scale. What one reviewer may find aesthetically pleasing, functional, or engaging, another reviewer may not. To account for these differences, the average of the 2 scores was used as the final score for each domain. The temporal relevancy of the results from this study may also be considered a limitation, despite the search being conducted less than 12 months ago because unlike traditional literature where the content remains consistent and unchanged once published, apps are extremely fluid, resulting in frequent updates and modifications to their contents and features. Despite these limitations, this study is the first of its kind and provides valuable real-world findings and implications that are highly relevant to this field as well as the greater audience. Future research should test the overall effectiveness of commercial apps designed to promote physical activity among pregnant women. Finally, research examining the accuracy of app content and the expertise of developers should also be of high priority.

In conclusion, the use of apps for physical activity advice and support in pregnancy is rising. Therefore, an understanding of their quality and inclusion of effective BCTs is required. This is the first study to investigate the quality of popular commercially available apps for promoting physical activity in women during pregnancy. The findings of moderate app quality, with the highest ratings for *functionality* and *aesthetics,* indicate that the apps are user-friendly. However, the low use of evidence-based BCTs for changing physical activity behavior in women during pregnancy indicates that the popular commercial apps currently available may not be effective at promoting physical activity behavior in the pregnant population. More effort needs to be placed on incorporating components most likely to influence behavior change, which is ultimately what they are developed for, while maintaining good functionality. Developers should continue to provide self-monitoring and social comparison and ensure that these components are engaging and effective. In addition, intention formation, goal setting, review of goals, and feedback on performance should be incorporated in new apps and new versions of existing apps.

## Appendix

Multimedia Appendix 1 Search strategy and search results.

Multimedia Appendix 2 App characteristics.

Multimedia Appendix 3 Mobile App Rating Scale categories.

Multimedia Appendix 4 Mean Mobile App Rating Scale scores and interrater reliability.

Multimedia Appendix 5 Behavior change techniques and interrater reliability.

## Abbreviations

BCT: behavior change technique
Kα: Krippendorff’s alpha
MARS: Mobile App Rating Scale
