# Supplementary material for: Quality, Features, and Presence of Behavior Change Techniques in Mobile Apps Designed to Improve Physical Activity in Pregnant Women: Systematic Search and Content Analysis
Source: JMIR Mhealth Uhealth. 2021 Apr 7;9(4):e23649. doi: 10.2196/23649 (PMC8060865; doi:10.2196/23649)
Supplement: Multimedia Appendix 1 [file mhealth_v9i4e23649_app1.doc]

**Multimedia Appendix 1: Search Strategy**

General search terms and categories used in the searches

| Search terms | Pregnancy exercises, pregnancy exercise, pregnancy fitness, pregnancy workout, pregnancy physical activity, pregnant exercises, pregnant exercise, pregnant fitness, pregnant workout, pregnant physical activity, prenatal exercises, prenatal exercise, prenatal fitness, prenatal workout, prenatal physical activity, postnatal, exercises, postnatal exercise, postnatal fitness, postnatal workout, and postnatal physical activity |
| --- | --- |
| Results listed in order of relevance | |
| Limits | Pregnant and/or postnatal women  Focus on exercise and/or physical activity  External/additional devices not required  Available in English |

**Apple iTunes**

Search timeframe: October 2018 - February 2019

Results by search term:

| Search Term | Total results (n) | Results after applying limits (n) |
| --- | --- | --- |
| Pregnancy exercises | 212 | 38 |
| Pregnancy exercise | 163 | 55 |
| Pregnancy fitness | 207 | 43 |
| Pregnancy workout | 208 | 68 |
| Pregnancy physical activity | 0 | 0 |
| Pregnant exercises | 27 | 21 |
| Pregnant exercise | 203 | 37 |
| Pregnant fitness | 207 | 48 |
| Pregnant workout | 205 | 52 |
| Pregnant physical activity | 0 | 0 |
| Prenatal exercises | 191 | 47 |
| Prenatal exercise | 46 | 30 |
| Prenatal fitness | 186 | 47 |
| Prenatal workout | 174 | 45 |
| Prenatal physical activity | 0 | 0 |
| Postnatal exercises | 25 | 21 |
| Postnatal exercise | 25 | 21 |
| Postnatal fitness | 64 | 36 |
| Postnatal workout | 64 | 36 |
| Postnatal physical activity | 0 | 0 |
| TOTAL | 2,207 | 645 |

**Google Play**

Search timeframe: October 2018 - February 2019

Results by search term:

| Search Term | Total results (n) | Results after applying limits (n) |
| --- | --- | --- |
| Pregnancy exercises | 250 | 100 |
| Pregnancy exercise | 250 | 100 |
| Pregnancy fitness | 250 | 100 |
| Pregnancy workout | 250 | 100 |
| Pregnancy physical activity | 250 | 100 |
| Pregnant exercises | 250 | 100 |
| Pregnant exercise | 250 | 100 |
| Pregnant fitness | 250 | 89 |
| Pregnant workout | 250 | 100 |
| Pregnant physical activity | 250 | 100 |
| Prenatal exercises | 250 | 100 |
| Prenatal exercise | 250 | 100 |
| Prenatal fitness | 250 | 99 |
| Prenatal workout | 250 | 100 |
| Prenatal physical activity | 250 | 100 |
| Postnatal exercises | 250 | 100 |
| Postnatal exercise | 250 | 48 |
| Postnatal fitness | 250 | 40 |
| Postnatal workout | 250 | 43 |
| Postnatal physical activity | 250 | 90 |
| TOTAL | 5,000 | 1,809 |
