# Supplementary material for: Quality, Features, and Presence of Behavior Change Techniques in Mobile Apps Designed to Improve Physical Activity in Pregnant Women: Systematic Search and Content Analysis
Source: JMIR Mhealth Uhealth. 2021 Apr 7;9(4):e23649. doi: 10.2196/23649 (PMC8060865; doi:10.2196/23649)
Supplement: Multimedia Appendix 2 [file mhealth_v9i4e23649_app2.doc]

**Multimedia Appendix 2: App Characteristics**

| **App Name** | **Developer** | **Version** | **Store** | **Cost** | **Rating** | **n Ratings** |
| --- | --- | --- | --- | --- | --- | --- |
| 9MonthsGuide | 9MonthsGuide Team | 3.2 | GooglePlay | Free | 4.5 | 463 |
| Fit to Be Pregnant | Creativelab | 1 | GooglePlay | Free | 5 | 1 |
| Get Parenting Pregnancy Tips. | BabyChakra-Indian and Parenting Tips | 7.4 | GooglePlay | Free | 4.7 | 13,000 |
| How to Get Pregnant Fast | Dub Apps | 1.0.3 | GooglePlay | Free | 4.4 | 7 |
| I'm Pregnant - Pregnancy Tracker | BabyJoyApp | 4 | GooglePlay | Free | 4.5 | 32 |
| iMum - Pregnancy & Fertility | OB Science S.r.l. | 3.5.3 | AppStore | Free | 5 | 8 |
| Kegel Exercises | Amila | 1.2.21 | GooglePlay | Free | 4.9 | 8,000 |
| MWM | Swide Srls | 1.1 | GooglePlay | Free | 5 | 4 |
| Pregnacise - Pregnancy Exercise App | Hungrydog Media Ltd | 1 | AppStore | $2.99 | 4.5 | 2 |
| Pregnancy + | Health and Parenting ltd | 1.1 | AppStore | Free | 4.5 | 5,432 |
| Pregnancy Guide | ARVIRA DEV | 3 | GooglePlay | Free | 4.5 | 5,000 |
| Pregnancy Health | HAYATY | 1.2 | GooglePlay | Free | 4.7 | 3 |
| Pregnancy Tips Offline | MakeMySystem | 2.1.11 | GooglePlay | Free | 4.7 | 650 |
| Pregnancy Tracker & Countdown | Timeline OOO | 1.88 | AppStore | Free | 4.6 | 798 |
| Pregnancy Week by Week Tracker | PregBuddy | 2.8.8 | GooglePlay | Free | 4.7 | 2,202 |
| Pregnancy Workouts - Baby2Body | Baby2Body Limited | 5.3.1 | AppStore | Free | 5 | 5 |
| Pregnant Mom, Baby and Toddler | Angry Panda Studio | 10 | GooglePlay | Free | 5 | 1 |
| Yoga for Pregnant Women | funentertainmentapps | 3 | GooglePlay | Free | 4.5 | 12 |
| Yoggy: Prenatal workout & Yoga | Millefeuille Agency | 1.5 | AppStore | Free | 4.5 | 8 |
