# Supplementary material for: Quality, Features, and Presence of Behavior Change Techniques in Mobile Apps Designed to Improve Physical Activity in Pregnant Women: Systematic Search and Content Analysis
Source: JMIR Mhealth Uhealth. 2021 Apr 7;9(4):e23649. doi: 10.2196/23649 (PMC8060865; doi:10.2196/23649)
Supplement: Multimedia Appendix 3 [file mhealth_v9i4e23649_app3.doc]

**Multimedia Appendix 3: MARS Categories**

| **App Name** | **MARS *Focus Points*** | **MARS *Theoretical Background/Strategies*** | **MARS *Technical Aspects*** |
| --- | --- | --- | --- |
| 9MonthsGuide | n = 3 Behaviour change Physical health Mindfulness/Meditation/Relaxation | n = 3 Information/Education Advice/Tips/Strategies/Skills training CBT-Behavioural (positive events) | n = 2 Has an app community Sends reminders |
| Fit to Be Pregnant | n = 3 Increased happiness/Well-being Behaviour change Physical health | n = 3 Information/Education Advice/Tips/Strategies/Skills training CBT - Behavioural (positive events) |  |
| Get Parenting Pregnancy Tips. | n = 3 Behaviour change Increase happiness/Well-being Physical health | n = 2 Information/Education Advice/Tips/Strategies/Skills training | n = 5 Has an app community Allows sharing (Facebook, Twitter etc) Sends reminders Requires login  Allows password protection |
| How to Get Pregnant Fast | n = 4 Increase happiness/Well-being Anxiety/Stress Relationships Physical health | n = 3  Information/Education Advice/Tips/Strategies/Skills training Mindfulness/Meditation |  |
| I'm Pregnant - Pregnancy Tracker | n = 1 Other (Tracks weight gain and baby growth; Uses timer and tracks level completion; Information on exercise, cost of pregnancy, what to take to hospital, and relaxation music) | n = 5 Information/Education Monitoring/Tracking Advice/Tips/Strategies/Skills training Relaxation Other (tracking of level completion and timing) |  |
| iMum - Pregnancy & Fertility | n = 5 Mindfulness/Meditation/Relaxation Behaviour change Goal setting Entertainment Physical health | n = 9 Assessment Feedback Information/Education Monitoring/Tracking Goal setting Relaxation Advice/Tips/Strategies/Skills training CBT - Behavioural (positive events) Mindfulness/Meditation | n = 2 Has an app community Sends reminders |

| **App Name** | **MARS *Focus Points*** | **MARS *Theoretical Background/Strategies*** | **MARS *Technical Aspects*** |
| --- | --- | --- | --- |
| Kegel Exercises | n = 2 Behaviour change Physical health | n = 3 Information/Education Monitoring/Tracking Advice/Tips/Strategies/Skills training | n = 1 Sends reminders |
| MWM | n = 5 Mindfulness/Meditation/Relaxation Anxiety/Stress Behaviour change Physical health Other (Posture) | n = 2 Information/Education Advice/Tips/Strategies/Skills training | n = 5 Allows sharing (Facebook, Twitter, etc.) Has an app community Allows password protection Requires login Needs web access to function |
| Pregnacise - Pregnancy Exercise App | n = 2 Behaviour change Physical health | n = 2 Information/Education Advice/Tips/Strategies/Skills training | n = 1 Has an app community |
| Pregnancy + | n = 2 Behaviour change  Physical health | n = 3 Feedback Information/Education Advice/Tips/Strategies/Skills training | n = 1 Allows sharing (Facebook, Twitter, etc.) |
| Pregnancy Guide | n = 1 Physical health | n = 1 Information/Education | n = 1 Allows sharing (Facebook, Twitter, etc) |
| Pregnancy Health | n = 1 Physical health | n = 2 Information/Education Advice/Tips/Strategies/Skills training | n = 1 Allows sharing (Facebook, Twitter, etc) |
| Pregnancy Tips Offline | n = 8 Increase happiness/Well-being Reduce negative emotions Anxiety/Stress Mindfulness/Meditation/Relaxation Behaviour change Goal setting Physical health Other (Health education) | n = 6 Information/Education Monitoring/Tracking Goal setting Advice/Tips/Strategies/Skills training Mindfulness/Meditation Relaxation | n = 1 Sends reminders |
| Pregnancy Tracker & Countdown | n = 5 Mindfulness/Meditation/Relaxation Physical health Behaviour change Depression Anxiety/Stress | N = 6 Information/Education Monitoring/Tracking Advice/Tips/Strategies/Skills training CBT - Behavioural (positive events) Mindfulness/Meditation Relaxation | n = 5 Allows sharing (Facebook, Twitter, etc.) Has an app community Allows password protection Requires login Sends reminders |

| **App Name** | **MARS *Focus Points*** | **MARS *Theoretical Background/Strategies*** | **MARS *Technical Aspects*** |
| --- | --- | --- | --- |
| Pregnancy Week by Week Tracker | n = 6 Increase happiness/Well-being Mindfulness/Meditation/Relaxation Reduce negative emotions Behaviour change Goal setting Physical health | n = 6 Information/Education Monitoring/tracking Goal setting Advice/Tips/Strategies/Skills training Mindfulness/Meditation Relaxation | n = 4 Has an app community Sends reminders Requires login Needs web access to function |
| Pregnancy Workouts - Baby2Body | N = 5 Reduce negative emotions Behaviour change Goal setting Physical health  Other (Scheduling support) | n = 3 Information/Education  Monitoring/Tracking Advice/Tips/Strategies/Skills training | n = 1 Allows sharing (Facebook, Twitter, etc) |
| Pregnant Mom, Baby and Toddler | n = 3 Behaviour change Physical health Other (intervention on exercise & pregnancy hidden in weekly tips at week 14) | n = 2 Information/Education Advice/Tips/Strategies/Skills training |  |
| Yoga for Pregnant Women | n= 3 Increased happiness/Well-being Behaviour change Physical health | n = 3 Information/Education Mindfulness/Meditation Relaxation | n = 2 Allows sharing (Facebook, Twitter, etc)  Sends reminders |
| Yoggy: Prenatal workout & Yoga | n = 5  Mindfulness/Meditation/Relaxation  Increase happiness/Well-being Anxiety/Stress Reduce negative emotions Physical health | n = 4 Information/Education Advice/Tips/Strategies/Skills training Mindfulness/Meditation Relaxation | n = 1 Sends reminders |

Abbreviations: MARS = Mobile Application Rating Scale.
