# Supplementary material for: Quality, Features, and Presence of Behavior Change Techniques in Mobile Apps Designed to Improve Physical Activity in Pregnant Women: Systematic Search and Content Analysis
Source: JMIR Mhealth Uhealth. 2021 Apr 7;9(4):e23649. doi: 10.2196/23649 (PMC8060865; doi:10.2196/23649)
Supplement: Multimedia Appendix 4 [file mhealth_v9i4e23649_app4.doc]

**Multimedia Appendix 4: Mean MARS Scores and Inter-rater Reliability**

| **App Name** | **Engagement** | **Functionality** | **Aesthetics** | **Information** | **Total  MARS Quality** | **Subjective Quality** | **Likelihood of Behavioural Impact** |
| --- | --- | --- | --- | --- | --- | --- | --- |
| 9MonthsGuide | 3.4 | 3.9 | 3.7 | 2.9 | 3.5 | 2.8 | 2.2 |
| Fit to Be Pregnant | 2.4 | 4.1 | 3 | 3 | 3.1 | 2 | 2.6 |
| Get Parenting Pregnancy Tips. | 4 | 4.4 | 4.2 | 3.2 | 3.9 | 2.9 | 2.7 |
| How to Get Pregnant Fast | 2.5 | 4.3 | 3.3 | 3.2 | 3.3 | 2.1 | 2.8 |
| I'm Pregnant - Pregnancy Tracker | 3 | 4.6 | 4 | 2.8 | 3.6 | 2.9 | 2.2 |
| iMum - Pregnancy & Fertility | 3.9 | 4.6 | 4.5 | 3.6 | 4.2 | 3.2 | 2.5 |
| Kegel Exercises | 3.2 | 4.4 | 4 | 4 | 3.9 | 2.9 | 2.5 |
| MWM | 2.7 | 3.6 | 3.2 | 2.7 | 3 | 2.1 | 2.8 |
| Pregnacise - Pregnancy Exercise App | 2.4 | 4.6 | 4.2 | 3.2 | 3.6 | 3 | 2.8 |
| Pregnancy + | 2.5 | 3.8 | 2.7 | 2.6 | 2.9 | 1.8 | 3.7 |
| Pregnancy Guide | 1.2 | 3.3 | 2.3 | 2.8 | 2.4 | 1.8 | 2.2 |
| Pregnancy Health | 1.6 | 3.8 | 3 | 3.2 | 2.9 | 2 | 2.7 |
| Pregnancy Tips Offline | 4.1 | 5 | 4.4 | 3.8 | 4.3 | 3.3 | 1.6 |
| Pregnancy Tracker & Countdown | 4.1 | 4.4 | 4.2 | 3.7 | 4.1 | 3.8 | 3.8 |
| Pregnancy Week by Week Tracker | 4.5 | 4.3 | 4.4 | 3.5 | 4.2 | 3.3 | 3.2 |
| Pregnancy Workouts - Baby2Body | 3 | 4.7 | 4.1 | 3.7 | 3.9 | 2.5 | 1.7 |
| Pregnant Mom, Baby and Toddler | 2.3 | 4.8 | 3.5 | 3.1 | 3.4 | 2 | 1.6 |
| Yoga for Pregnant Women | 3.8 | 3.3 | 3.3 | 2.8 | 3.3 | 1.5 | 2.7 |
| Yoggy: Prenatal workout & Yoga | 2.5 | 4.3 | 4.1 | 2.8 | 3.4 | 2.4 | 1.9 |
| ***m*Kalpha** |  |  |  |  |  |  | **0.3** |

Abbreviations: MARS = Mobile Application Rating Scale; *m*Kalpha = mean Krippendorff’s alpha
