# Supplementary material for: Quality, Features, and Presence of Behavior Change Techniques in Mobile Apps Designed to Improve Physical Activity in Pregnant Women: Systematic Search and Content Analysis
Source: JMIR Mhealth Uhealth. 2021 Apr 7;9(4):e23649. doi: 10.2196/23649 (PMC8060865; doi:10.2196/23649)
Supplement: Multimedia Appendix 5 [file mhealth_v9i4e23649_app5.doc]

**Multimedia Appendix 5: BCTs and Inter-rater Reliability**

| **App Name** | **Provide information about behaviour-health link** | **Provide information on consequences** | **Provide information about other’s approval** | **Prompt intention formation** | **Prompt barrier identification** | **Provide general encouragement** | **Set graded tasks** | **Provide instructions** | **Model or demonstrate the behaviour** | **Prompt specific goal setting** | **Prompt review of behavioural goals** | **Prompt self-monitoring of behaviour** | **Provide feedback on performance** |
| --- | --- | --- | --- | --- | --- | --- | --- | --- | --- | --- | --- | --- | --- |
| 9MonthsGuide | 0 | 1 | 0 | 0 | 0 | 0 | 0 | 1 | 0 | 0 | 0 | 0 | 0 |
| Fit to Be Pregnant | 1 | 1 | 0 | 0 | 0 | 0 | 0 | 1 | 0 | 0 | 0 | 0 | 0 |
| Get Parenting Pregnancy Tips. | 0 | 1 | 0 | 0 | 0 | 0 | 0 | 1 | 1 | 0 | 0 | 0 | 0 |
| How to Get Pregnant Fast | 0 | 1 | 0 | 0 | 0 | 0 | 0 | 1 | 0 | 0 | 0 | 0 | 0 |
| I'm Pregnant - Pregnancy Tracker | 0 | 0 | 0 | 0 | 0 | 0 | 0 | 1 | 0 | 0 | 0 | 1 | 0 |
| iMum - Pregnancy & Fertility | 0 | 1 | 0 | 0 | 0 | 1 | 1 | 1 | 1 | 0 | 0 | 1 | 0 |
| Kegel Exercises | 0 | 1 | 0 | 0 | 0 | 0 | 0 | 1 | 1 | 0 | 0 | 1 | 0 |
| MWM | 0 | 0 | 0 | 0 | 0 | 0 | 0 | 1 | 1 | 0 | 0 | 0 | 0 |
| Pregnacise - Pregnancy Exercise App | 0 | 1 | 0 | 0 | 0 | 0 | 0 | 1 | 1 | 0 | 0 | 0 | 0 |
| Pregnancy + | 0 | 1 | 0 | 0 | 0 | 0 | 0 | 1 | 1 | 0 | 1 | 1 | 0 |
| Pregnancy Guide | 0 | 1 | 0 | 0 | 0 | 0 | 0 | 0 | 0 | 0 | 0 | 0 | 0 |
| Pregnancy Health | 1 | 1 | 0 | 0 | 0 | 0 | 0 | 1 | 0 | 0 | 0 | 0 | 0 |
| Pregnancy Tips Offline | 0 | 1 | 0 | 0 | 0 | 0 | 0 | 1 | 0 | 0 | 0 | 0 | 0 |
| Pregnancy Tracker & Countdown | 0 | 1 | 0 | 0 | 0 | 1 | 0 | 1 | 1 | 0 | 0 | 1 | 0 |
| Pregnancy Week by Week Tracker | 0 | 1 | 0 | 0 | 0 | 0 | 1 | 1 | 1 | 1 | 0 | 1 | 0 |
| Pregnancy Workouts - Baby2Body | 1 | 1 | 0 | 0 | 0 | 0 | 0 | 1 | 0 | 0 | 0 | 1 | 0 |
| Pregnant Mom, Baby and Toddler | 0 | 1 | 0 | 0 | 0 | 0 | 0 | 1 | 0 | 0 | 0 | 0 | 0 |
| Yoga for Pregnant Women | 0 | 1 | 0 | 0 | 0 | 0 | 0 | 1 | 1 | 0 | 0 | 0 | 0 |
| Yoggy: Prenatal workout & Yoga | 0 | 1 | 0 | 0 | 0 | 0 | 0 | 1 | 1 | 0 | 0 | 0 | 0 |

| **App Name** | **Provide contingent rewards** | **Teach to use prompts or cues** | **Agree on behavioural contract** | **Prompt practice** | **Use follow-up prompts** | **Provide opportunities for social comparison** | **Plan social support or social change** | **Prompt identification as a role model** | **Prompt self-talk** | **Relapse prevention** | **Stress management** | **Motivational interviewing** | **Time management** | **Frequency of techniques** |
| --- | --- | --- | --- | --- | --- | --- | --- | --- | --- | --- | --- | --- | --- | --- |
| 9MonthsGuide | 0 | 0 | 0 | 1 | 1 | 1 | 0 | 0 | 0 | 0 | 0 | 0 | 0 | **5** |
| Fit to Be Pregnant | 0 | 0 | 0 | 0 | 0 | 0 | 0 | 0 | 0 | 0 | 0 | 0 | 0 | **3** |
| Get Parenting Pregnancy Tips. | 0 | 0 | 0 | 1 | 1 | 1 | 0 | 0 | 0 | 0 | 0 | 0 | 0 | **6** |
| How to Get Pregnant Fast | 0 | 0 | 0 | 0 | 0 | 0 | 0 | 0 | 0 | 0 | 1 | 0 | 0 | **3** |
| I'm Pregnant - Pregnancy Tracker | 0 | 0 | 0 | 0 | 0 | 0 | 0 | 0 | 0 | 0 | 1 | 0 | 0 | **3** |
| iMum - Pregnancy & Fertility | 0 | 0 | 0 | 1 | 1 | 0 | 0 | 0 | 0 | 0 | 1 | 0 | 0 | **9** |
| Kegel Exercises | 0 | 0 | 0 | 1 | 1 | 0 | 0 | 0 | 0 | 0 | 0 | 0 | 0 | **6** |
| MWM | 0 | 0 | 0 | 0 | 0 | 0 | 0 | 0 | 0 | 0 | 0 | 0 | 0 | **2** |
| Pregnacise - Pregnancy Exercise App | 0 | 0 | 0 | 0 | 0 | 0 | 0 | 0 | 0 | 0 | 0 | 0 | 0 | **3** |
| Pregnancy + | 0 | 0 | 0 | 0 | 0 | 1 | 0 | 0 | 0 | 0 | 0 | 0 | 0 | **6** |
| Pregnancy Guide | 0 | 0 | 0 | 0 | 0 | 1 | 0 | 0 | 0 | 0 | 0 | 0 | 0 | **2** |
| Pregnancy Health | 0 | 0 | 0 | 0 | 0 | 1 | 0 | 0 | 0 | 0 | 0 | 0 | 0 | **4** |
| Pregnancy Tips Offline | 0 | 0 | 0 | 0 | 0 | 0 | 0 | 0 | 0 | 0 | 0 | 0 | 0 | **2** |
| Pregnancy Tracker & Countdown | 0 | 0 | 0 | 1 | 1 | 1 | 0 | 0 | 0 | 0 | 1 | 0 | 0 | **9** |
| Pregnancy Week by Week Tracker | 0 | 0 | 0 | 1 | 1 | 1 | 0 | 0 | 0 | 0 | 1 | 0 | 0 | **10** |
| Pregnancy Workouts - Baby2Body | 0 | 0 | 0 | 0 | 0 | 1 | 0 | 0 | 0 | 0 | 0 | 0 | 0 | **5** |
| Pregnant Mom, Baby and Toddler | 0 | 0 | 0 | 0 | 0 | 0 | 0 | 0 | 0 | 0 | 0 | 0 | 0 | **2** |
| Yoga for Pregnant Women | 0 | 0 | 0 | 0 | 0 | 0 | 0 | 0 | 0 | 0 | 1 | 0 | 0 | **4** |
| Yoggy: Prenatal workout & Yoga | 0 | 0 | 0 | 1 | 1 | 0 | 0 | 0 | 0 | 0 | 1 | 0 | 0 | **6** |
| **Percentage agreement** |  |  |  |  |  |  |  |  |  |  |  |  |  | **95%** |
| **Kalpha** |  |  |  |  |  |  |  |  |  |  |  |  |  | **0.85** |

Abbreviations: BCT = Behavioural Change Techniques; Kalpha = Krippendorff’s alpha
